# Good Outcome after Digoxin Toxicity Despite Very High Serum Potassium Level

**DOI:** 10.5812/kowsar.20741804.2238

**Published:** 2011-09-15

**Authors:** F Zand, S Asadi, P Katibeh

**Affiliations:** 1 Department of Anesthesiology, Shiraz University of Medical Sciences, Shiraz, Iran; 2 Department of Pediatrics, Shiraz University of Medical Sciences, Shiraz, Iran

**Keywords:** Digoxin toxicity, Serum potassium level

Dear Editor,

Digoxin, as a cardiac glycoside extracted from the Digitalis lanata[1] is widely used in the treatment of different heart diseases, such as atrial fibrillation, atrial flutter and heart failure.[2] Its effect is to increase myocardial contractility while mildly prolonging the duration of contraction. The result is decreased heart rate, increased blood pressure and stroke volume, leading to increased tissue perfusion, improved myocardial function and hemodynamics.[2] Digoxin interacts with verapamil, amiodarone, and erythromycin, leading to increase in its plasma level.[3] Arrhythmogenesis and atrio-ventricular conduction arrhythmias such as paroxysmal atrial tachycardia with A-V block are diagnostic for digoxin toxicity.[4] Without the use of digoxin-specific Fab fragments, in a recent review, mortality after digoxin toxicity have been reported to be zero in patients with acute digoxin toxicity and potassium levels less than 5.0 meq/dl, 50% in potassium levels between 5.0 and 5.5, and 100 percent in those with potassium levels above 5.5 meq/dl.[5] Serum potassium levels above 5.0 meq/dl in patients with acute cardiac digoxin toxicity has been proposed as an absolute indication for digoxin-specific Fab fragments.[5] We present a case of acute digoxin toxicity with high potassium level and good outcome despite unavailability of digoxin-specific Fab fragments.

**Fig. 1:**
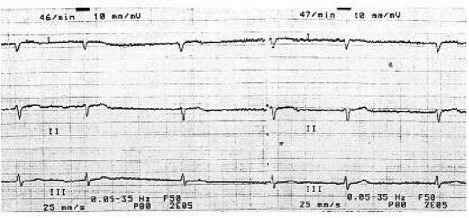
Atrial fibrillation. EKG of the Patient concomitant to “digoxin toxicity”.

The patient was a 75 year-old lady, who was admitted in intensive care unit (ICU) for management of respiratory failure after pulmonary emboli and pulmonary arterial hypertension. She had tracheostomy tube due to prolonged mechanical ventilation and her Glascow Coma Score was 11. The patient was receiving digoxin 0.25 mg daily via nasogastric tube for treatment of right sided heart failure. In ICU course due to feeding intolerance and decreased gastric motility, intravenous erythromycin (500 mg every 6 hours) was started as a prokinetic agent. Two days later, probably as a complication of drug interaction (digoxin and erythromycin), she developed vomiting, diarrhea, bradycardia, atrial fibrillation in favor of digoxin toxicity (Figure 1).

In her concomitant lab-data, serum potassium was reported 6.6 meq/l and repeated sample confirmed hyperkalemia (6.5 meq/lit); no hemolysis was reported and samples were taken from central venous line. So digoxin was hold and its serum level was checked with HPLC (High Performance Liquid Chromatography) method which was 3.2 ng/ml, in favor of digoxin toxicity (serum more than 2 ng/ml is considered as toxic range).

After holding digoxin and supportive management (close EKG monitoring, holding maintenance intravenous KCl, and administration of 50% dextroseregular insulin intravenously to control hyperkalemia) but without the use of 'digoxin-specific Fab fragments' (unfortunately digifab was unavailable at that time), signs and symptoms of digoxin toxicity was relieved and the patient survived despite concomitant high serum potassium 6.6 meq/l.

Digoxin effect is to increase intracellular amounts of Ca(2+) ions and K+ conductance. Other effects of this drug are increase in the refractory period of sinoatrial and AV nodes, decreasing it in the atria and ventricles, and so increase in excitability. It also decreases the conduction of electrical impulses through the AV node by vagal stimulation leading to negative chronotropic effect.[6]

Digoxin decreases the function of α-subunit of the Na(+)/K(+) ATPase pump in the membranes of heart myocytes by binding to it. So the level of the sodium ions in the myocytes increases, and as a consequence of sodium/calcium exchanger function intracellular calcium ions rise. Increased amount of Ca(2+) storing in the sarcoplasmic reticulum leads to increased contractility of the heart.[7] Digoxin is used in congestive heart failure, especially in patients with refractory symptoms along with diuretic and ACE inhibitor treatment, but it is ineffective at decreasing morbidity and mortality while improving the quality of life.[2]

Safe therapeutic plasma level is 1-2.6 nmol/l. Digoxin plasma level should be checked in the setting of questioned toxicity or ineffectiveness. Plasma potassium levels should be monitored frequently.[2] Due to narrow therapeutic index, the dose-dependent adverse effects (rare if plasma digoxin concentration is <0.8 μg/l) are common.[8] Hypokalemia increases the risk because digoxin competes with K(+) for binding to the Na+/K+ ATPase pump.[9] In overdose, supportive care should be undertaken. Digoxin antidote is digibind and digifab which is given in life threatening arrhythmias or malignant hyperkalemia which defines as unavoidably increasing potassium level owing to paralysis of the cell membrane bound ATPasedependent Na/K pumps.[10] EKG findings are increased PR interval, decreased QT interval, inverted T wave and ST depression. Other changes are AV junctional rhythm and ectopic beats (bigemminy) leading to ventricular tachycardia and fibrillation.[2]

Digoxin toxicity should be considered when deciding to start any new medication in a patient on digoxin therapy especially in ICU settings where poly pharmacy is not uncommon. Patients with acute cardiac glycoside toxicity and hyperkalemia especially those with serum potassium level above 5.5 meq/lit have a grave prognosis, but early and aggressive supportive therapy my significantly improve the outcome despite unavailability of digifab.
